# Thermal and solute aspects among two viscosity models in synovial fluid inserting suspension of tri and hybrid nanomaterial using finite element procedure

**DOI:** 10.1038/s41598-022-23271-0

**Published:** 2022-12-14

**Authors:** Umar Nazir, Muhammad Sohail, Poom Kumam, Yasser Elmasry, Kanokwan Sitthithakerngkiet, Mohamed R. Ali, Muhammad Jahangir Khan, Ahmed M. Galal

**Affiliations:** 1 Department of Mathematics, Faculty of Science, Khon Kaen University, Khon Kaen, 40002 Thailand; 2 Department of Mathematics, Khwaja Fareed University of Engineering & Information Technology, Rahim Yar Khan, 64200 Pakistan; 3 Center of Excellence in Theoretical and Computational Science (TaCS-CoE) & KMUTT Fixed Point Research Laboratory, Room SCL 802 Fixed Point Laboratory, Science Laboratory Building, Departments of Mathematics, Faculty of Science, King Mongkut’s University of Technology Thonburi (KMUTT), 126 Pracha-Uthit Road, Bang Mod, Thung Khru, Bangkok, 10140 Thailand; 4 Department of Medical Research, China Medical University Hospital, China Medical University, Taichung, 40402 Taiwan; 5 Department of Mathematics, Faculty of Science, King Khalid University, P.O. Box 9004, Abha, 61466 Saudi Arabia; 6 Department of Mathematics, Faculty of Science, Mansoura University, Mansoura, 35516 Egypt; 7 Intelligent and Nonlinear Dynamic Innovations Research Center, Department of Mathematics, Faculty of Applied Science, King Mongkut’s University of Technology North Bangkok (KMUTNB), 1518, Wongsawang, Bangsue, Bangkok, 10800 Thailand; 8 Faculty of Engineering and Technology, Future University in Egypt, New Cairo, 11835 Egypt; 9 Department of Advance Materials and Technologies, Faculty of Materials Engineering, Silesian University of Technology, 44-100 Gliwice, Poland; 10 Mechanical Engineering Department, College of Engineering, Prince Sattam Bin Abdulaziz University, Wadi Addawaser, 11991 Saudi Arabia; 11 Production Engineering and Mechanical Design Department, Faculty of Engineering, Mansoura University, P.O 35516, Mansoura, Egypt

**Keywords:** Mathematics and computing, Nanoscience and technology

## Abstract

Inclusion of nanoparticles boosts thermal performance and is essential for thermal transport. The current investigation has been made to conduct research on heat mass transport in synovial material with the mixing of hybrid and tri-hybrid comprising variable viscosity past over a heated surface having constant density and a steady environment. The conservation laws have been considered in the presence of Lorentz force, heat generation/absorption, modified heat and mass fluxes together with chemical reaction. The mathematical model is developed in Cartesian coordinate in the form of coupled partial differential equation (PDEs). The derived PDEs are simplified by a boundary layer approach (BLA) and reduced PDEs have been converted into ordinary differential equation (ODEs) using scaling group Similarity transformation. The converted ODEs are highly nonlinear and have been solved numerically by finite elements scheme (FES). The used scheme is effective for nonlinear problem and can be frequently utilized to tackle nonlinear problems arising in mathematical physics.

## Introduction

Researchers got much attention on studying nanoparticles to boost thermal performance. Nanoparticles are immersed in different materials (base fluid) to improve thermal performance. Nanomaterial plays a vital role in the treatment of cancer therapy and is frequently used in industries in different physical processes. Several studies have been conducted on nanomaterial for thermal and mass transportation. For instance, Nazir et al.^1^ estimated comparative consequences among variable and constant viscosities in Carreau fluid over a stretchable frame. They have used FEM to know numerical results of model related to non-Fourier’s along with variable viscosity. Sohail et al.^2^ performed model of Sutterby fluid inserting suspension of nanomaterial along with thermal radiation past stretching surface in the presence of variable fluidic properties. They have used a numerical approach to conduct consequences. Nazir et al.^3^ conducted numerical impacts of Carreau–Yasuda martial in mass species and thermal energy inserting hybrid nanomaterial under the occurrence of thermal properties numerical implemented by finite element approach. Sohail et al.^4^ computed boundary layer flow regarding Carreau martial in heat energy in a disk considering bio-convection phenomena. They have implemented numerical method to investigate numerical results. Nazir et al.^5^ performed the thermal enhancement of ternary hybrid nanoparticles in Sisko martial involving heat generation over stretchable cylinder. They implemented FEM and include the tri-hybrid nanomaterial was significant the best performer to enhance heat energy. Imran et al.^6^ investigated the performance of shapes impacts into hybrid nanoparticles past flat frame considering solar collector. Imran et al.^7^ studied the impacts of thermal radiation and magnetic field including variable fluidic properties and slip conditions past a vertical heated frame. Danish et al.^8^ conducted numerical consequences of Williamson liquid inserting nanoparticles in the presence of thermal radiation and activation energy. Chen et al.^9^ discussed enhancement regarding heat energy in Maxwell liquid in the presence of titanium oxide and alumina oxide in rotating disks. Sohail et al.^10^ studied features of non-Newtonian liquid in heat energy using Joule heating and heat flux approach. Mebarek-Oudina et al.^11^ studied two heat energy sources in mixed convection phenomena in a trapezoidal cavity using moving walls. Chabani et al.^12^ investigate heat energy enhancement in triangular enclosure considering suspension regarding hybrid nanoparticles involving elliptic obstacle. Fares et al.^13^ investigated the performance of entropy generation in magnetized flow in the presence of Darcy–Forchheimer model using base liquid (silver and water) in a square enclosure. Abo-Dahab et al.^14^ securitized MHD features in Casson liquid considering nanoparticles involving dual behaviors of surface (suction and injection) past stretchable surface. Mebarek-Oudina et al.^15^ discussed applications of nanoparticles in heat energy mechanism using various enclosures. Dhif et al.^16^ investigated the performance of hybrid nanofluids in solar collectors in this study. Zamzari et al.^17^ studied mixed convection in a vertically heated channel to determine various aspects of entropy production. Using this method, multiple aspects of entropy production were examined to determine how they were affected. Li et al.^18^ determined the flow and thermal properties of a Darcy–Forchheimer fluid using a non-Fourier method and a Prandtl method. To achieve this objective, nanoparticles were incorporated into a Darcy–Forchheimer motion. Klazly et al.^19^ studied comparative consequences among non-Newtonian for single-phase and Newtonian for single-phase containing nanoparticles in the presence of two-phase models in backward-facing step. Klazly et al.^20^ developed empirical equation to visualize viscosity of nanofluid involving empirical and theoretical results. Bognár et al.^21^ investigated comparative consequences among fluid dynamics and similarity in Blasius flow considering nanoparticles. Thriveni and Mahanthesh^22^ discussed features based on convective heat transfer in hybrid nanofluid considering in two cylinders. Mackolil and Mahanthesh^23^ estimated thermal energy associated with Marangoni approach in hybrid nanofluid including sensitivity analysis. Mahanthesh^24^ discussed features regarding quadratic radiative flux containing nanoparticles in the presence of enhancement of kinematics regarding nanoparticles. Mahanthesh et al.^25^ performed new investigation based on quadratic Boussinesq approximation and quadratic radiation involving nanofluid. Shruthy and Mahanthesh^26^ computed Rayleigh-Bénard in rheology of Casson liquid inserting suspension of hybrid nanoparticles. Shalini and Mahanthesh^27^ discussed performance of dusty nanoparticles in Newtonian martial considering suspension of nanoparticles in the presence of coriolis force.

Comprehensive review on literature demonstrates that study on mass species and heat energy in the presence of Cattaneo-Christov heat flux model (CCHM) along with chemical reaction and heat absorption in synovial fluid is not investigated yet. Such complex model involving suspension of three types of nanoparticles considering two viscosity models is not studied yet. Additionally, hybrid nanomaterial and tri-hybrid nanomaterial are inserted in ethylene glycol. Present model is numerically solved by finite element method (FEM). Analysis is divided into five sections. Section one, section two, section three, section four and section five are based on literature review, mathematical procedure, numerical method and conclusions, etc.

## Mathematical procedure

**Figure 1:**
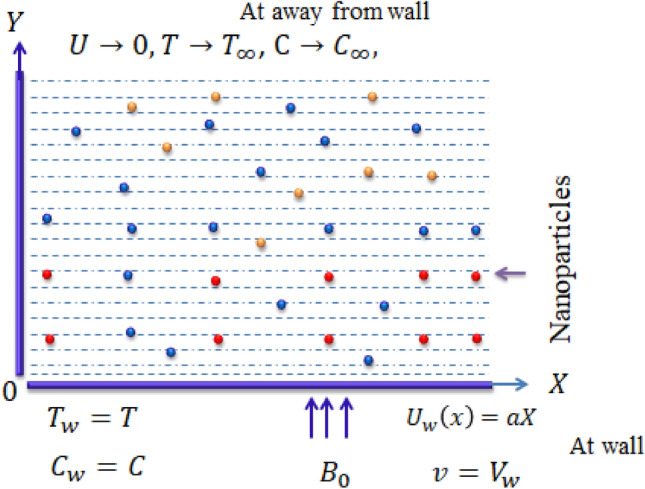
Geometry of current problem.

Model regarding mass and heat energy transport in synovial fluid is developed for constant density and steady flow past a stretching surface. Viscosity is considered as function temperature and concentration. Suspension of tri-hybrid nanoparticles into fluidic motion is made composite of silicon dioxide, titanium oxide and aluminum oxide in the presence of base liquid named ethylene glycol. Fluidic motion regarding various nanoparticles is produced with the help of wall velocity. Heat and energy solute particles are carried out using concept of non-Fourier’s law including heat generation/absorption and chemical reaction. Figure 1 captures flow and thermal transport in a geometrical view. The physical situation of the developed model is transformed into a system of PDEs, which is formulated.

The concept of BLA (boundary layer approximations) are used to achieve system of PDEs are

1 $$\frac{\partial u}{\partial X}+\frac{\partial v}{\partial Y}=0.$$

Momentum equation for Model-I which is

2 $$ \begin{aligned} \rho_{th} \left( {u\frac{\partial u}{{\partial X}} + v\frac{\partial v}{{\partial Y}}} \right) & = 2\mu_{th} \left[ {\begin{array}{*{20}c} {\frac{{\partial^{2} u}}{{\partial Y^{2} }} + 3mY^{2} \left( {\frac{\partial u}{{\partial Y}}} \right)^{2} \frac{{\partial^{2} u}}{{\partial Y^{2} }} + \alpha \frac{{\partial^{2} u}}{{\partial Y^{2} }}\phi + \alpha \frac{\partial u}{{\partial Y}}\frac{\partial \phi }{{\partial Y}}} \\ { + \alpha mY^{2} \left( {\frac{\partial u}{{\partial Y}}} \right)^{3} \frac{\partial \phi }{{\partial Y}} + 3m\gamma^{2} \phi \left( {\frac{\partial u}{{\partial Y}}} \right)^{2} \frac{{\partial^{2} u}}{{\partial Y^{2} }}} \\ \end{array} } \right] \\ & \quad - 2\mu_{th} \sigma_{th} B_{0}^{2} u. \\ \end{aligned} $$

Momentum equation for Model-II which is

3 $$ \begin{aligned} \rho_{th} \left( {u\frac{\partial u}{{\partial X}} + v\frac{\partial v}{{\partial Y}}} \right) & = 2\mu_{th} \left[ {\frac{{\partial^{2} u}}{{\partial Y^{2} }} - \frac{{\alpha Y^{2} }}{2}\left( {\frac{\partial u}{{\partial Y}}} \right)^{3} \frac{\partial \phi }{{\partial Y}} - \frac{{3\alpha Y^{2} }}{2}\phi \left( {\frac{\partial u}{{\partial Y}}} \right)^{2} \frac{{\partial^{2} u}}{{\partial Y^{2} }}} \right] \\ & - 2\mu_{th} \sigma_{th} B_{0}^{2} u. \\ \end{aligned} $$

4 $$ \begin{aligned} & u\frac{\partial T}{{\partial X}} + v\frac{\partial T}{{\partial Y}} + \gamma_{1} \left[ {\begin{array}{*{20}c} {u^{2} \frac{{\partial^{2} T}}{{\partial X^{2} }} + v^{2} \frac{{\partial^{2} T}}{{\partial Y^{2} }} + 2uv\frac{\partial T}{{\partial X\partial Y}} + \left( {u\frac{\partial u}{{\partial X}} + v\frac{\partial u}{{\partial Y}}} \right)\frac{\partial T}{{\partial X}}} \\ {\left( {u\frac{\partial v}{{\partial X}} + v\frac{\partial v}{{\partial Y}}} \right)\frac{\partial T}{{\partial Y}} - \frac{{Q_{0} }}{{\left( {\rho C_{p} } \right)_{th} }}\left( {u\frac{\partial u}{{\partial X}} + v\frac{\partial u}{{\partial x}}} \right)} \\ \end{array} } \right] \\ & \quad = \frac{{K_{th} }}{{\left( {\rho C_{p} } \right)_{th} }}\frac{{\partial^{2} T}}{{\partial Y^{2} }} - \frac{{Q_{0} }}{{\left( {\rho C_{p} } \right)_{th} }}\left( {T - T_{\infty } } \right), \\ \end{aligned} $$

5 $$ \begin{aligned} & u\frac{\partial C}{{\partial X}} + v\frac{\partial C}{{\partial Y}} + \gamma_{2} \left[ {\begin{array}{*{20}c} {u^{2} \frac{{\partial^{2} C}}{{\partial X^{2} }} + v^{2} \frac{{\partial^{2} C}}{{\partial Y^{2} }} + 2uv\frac{\partial C}{{\partial X\partial Y}} + \left( {u\frac{\partial u}{{\partial X}} + v\frac{\partial u}{{\partial Y}}} \right)\frac{\partial C}{{\partial X}}} \\ {\left( {u\frac{\partial v}{{\partial X}} + v\frac{\partial v}{{\partial Y}}} \right)\frac{\partial C}{{\partial Y}} - k_{1} \left( {u\frac{\partial C}{{\partial X}} + v\frac{\partial C}{{\partial x}}} \right)} \\ \end{array} } \right] \\ & \quad = D_{th} \frac{{\partial^{2} C}}{{\partial Y^{2} }} - k_{1} \left( {C - C_{\infty } } \right). \\ \end{aligned} $$

The required BCs of current analysis are

6 $$T\to {T}_{\infty }, u\to 0, C\to {C}_{\infty }:Y\to \infty , C={C}_{w}, u=aX, v=0, T={T}_{w}:Y=0.$$

Similarity variables are delivered as

7 $$v=-\frac{\partial \psi }{\partial X}, \theta =\frac{{T}_{\infty }-T}{{T}_{\infty }-{T}_{w}}, \eta ={\left(\frac{a}{{\nu }_{f}}\right)}^\frac{1}{2}Y,\phi =\frac{{C}_{\infty }-C}{{C}_{\infty }-{C}_{w}}, u= \frac{\partial \psi }{\partial Y}.$$

The Correlations of hybrid nanoparticles and nanoparticles are used and Table 1 contains the numerical values

**Table 1:** Thermal properties of hybrid nanoparticles with base fluid.

Particles	$k$	$\rho$	$\sigma$
${Al}_{2}{O}_{3}$	32.9	6310	$5.96\times {10}^{7}$
$Si{O}_{2}$	1.4013	2270	$3.5\times {10}^{6}$
$Ti{O}_{2}$	8.953	4250	$2.4\times {10}^{6}$
EG	0.144	884	$0.125\times {10}^{-11}$

8 $${\rho }_{th}=\left(1-{\varphi }_{1}\right)\left\{\left(1-{\varphi }_{2}\right)\left[\left(1-{\varphi }_{3}\right){\rho }_{f}+{\varphi }_{3}{\rho }_{3}\right]+{\varphi }_{2}{\rho }_{2}\right\}+{\varphi }_{1}{\rho }_{1},$$

9 $${\mu }_{th}=\frac{{\left(1-{\varphi }_{3}\right)}^{-2.5}{\mu }_{f}}{{\left(1-{\varphi }_{2}\right)}^{2.5}{\left(1-{\varphi }_{1}\right)}^{2.5}}, \frac{{K}_{hnf}}{{K}_{nf}}=\frac{{K}_{2}+2{K}_{nf}-2{\varphi }_{1}\left({K}_{nf}-{K}_{2}\right)}{{K}_{2}+2{K}_{nf}+{\varphi }_{2}\left({K}_{nf}-{K}_{2}\right)}, \frac{{\sigma }_{nf}}{{\sigma }_{f}}=\frac{{\sigma }_{3}\left(1+2{\varphi }_{3}\right)+{\varphi }_{f}\left(1-2{\varphi }_{3}\right)}{{\sigma }_{3}(1-{\varphi }_{3})+{\sigma }_{f}(1+{\varphi }_{3})},$$

10 $$\frac{{K}_{th}}{{K}_{hnf}}=\frac{-2{\varphi }_{1}\left({K}_{hnf}-{K}_{1}\right){K}_{1}+2{K}_{hnf}}{{K}_{1}+2{K}_{hnf}+{\varphi }_{1}\left({K}_{hnf}-{K}_{1}\right)}, \frac{{K}_{nf}}{{K}_{f}}=\frac{{K}_{3}+2{K}_{f}-2{\varphi }_{3}\left({K}_{f}-{K}_{3}\right)}{{K}_{3}+2{K}_{f}+{\varphi }_{3}\left({K}_{f}-{K}_{3}\right)},$$

11 $$\frac{{\sigma }_{th}}{{\sigma }_{hnf}}=\frac{{\sigma }_{1}\left(1+2{\varphi }_{1}\right)-{\varphi }_{hnf}\left(1-2{\varphi }_{1}\right)}{{\sigma }_{1}(1-{\varphi }_{1})+{\sigma }_{hnf}(1+{\varphi }_{1})}, \frac{{\sigma }_{hnf}}{{\sigma }_{nf}}=\frac{{\sigma }_{2}\left(1+2{\varphi }_{2}\right)+{\varphi }_{nf}\left(1-2{\varphi }_{2}\right)}{{\sigma }_{2}(1-{\varphi }_{2})+{\sigma }_{nf}(1+{\varphi }_{2})}.$$

A system in term of ODEs is derived as

12 $$ \begin{aligned} & 2\left[ {\left( {1 + \alpha \phi + mRewe^{2} F^{{\prime}{2}} + 3mRewe^{2} F^{{\prime}{2}} \phi } \right)F^{\prime\prime\prime} - \frac{{\nu_{f} }}{{\nu_{th} }}F^{{\prime}{2}} + \frac{{\nu_{f} }}{{\nu_{th} }}FF^{\prime\prime} + 2m\alpha \gamma^{2} F^{\prime\prime}\phi } \right] \\ & \quad + 2m\gamma^{2} F^{\prime\prime}\phi - \frac{{\sigma_{f} }}{{\sigma_{th} }}M^{2} F^{\prime} + 2mRewe^{2} F^{{\prime}{3}} \phi = 0, \\ \end{aligned} $$

13 $$  2\left[ {\left( {1 - 3\alpha Rewe^{2} F^{{\prime 2}} \phi } \right)F^{{\prime \prime \prime }} } \right] - \frac{{\nu _{f} }}{{\nu _{{th}} }}F^{{\prime 2}}  + \frac{{\nu _{f} }}{{\nu _{{th}} }}FF^{{\prime \prime }}  - \alpha Rewe^{2} F^{{\prime 3}} \phi  - \frac{{\sigma _{f} }}{{\sigma _{{th}} }}M^{2} F^{\prime }  = 0, $$

14 $$ \theta^{\prime\prime} + \frac{{k_{f} \left( {\rho C_{p} } \right)_{th} }}{{k_{th} \left( {\rho C_{p} } \right)_{f} }}PrF\theta^{\prime} - \delta_{1} \frac{{k_{f} \left( {\rho C_{p} } \right)_{th} }}{{k_{th} \left( {\rho C_{p} } \right)_{f} }}Pr\left[ {FF^{\prime}\theta^{\prime} + F^{2} \theta^{\prime\prime} + H_{t} F\theta^{\prime}} \right] + \frac{{k_{f} }}{{k_{th} }}PrH_{t} \theta = 0, $$

15 $$ \begin{aligned} & \phi^{\prime\prime} + \frac{{\left( {1 - \varphi_{2} } \right)^{ - 2.5} Sc}}{{\left( {1 - \varphi_{1} } \right)^{2.5} \left( {1 - \varphi_{3} } \right)^{2.5} }}F\phi^{\prime} - \delta_{2} \frac{{\left( {1 - \varphi_{2} } \right)^{ - 2.5} Sc}}{{\left( {1 - \varphi_{1} } \right)^{2.5} \left( {1 - \varphi_{1} } \right)^{2.5} }}\left[ {FF^{\prime}\phi^{\prime} + F^{2} \phi^{\prime\prime} + K_{c} F\phi^{\prime}} \right] \\ & \quad - \frac{{\left( {1 - \varphi_{2} } \right)^{ - 2.5} ScK_{c} }}{{\left( {1 - \varphi_{1} } \right)^{2.5} \left( {1 - \varphi_{1} } \right)^{2.5} }}\phi = 0. \\ \end{aligned} $$

Equation (6) is reduced into dimensionless form which is

16 $$\phi \left(\infty \right)=0,F\left(0\right)=0, \theta \left(0\right)=1, {F}^{{^{\prime}}}\left(0\right)=1, \theta \left(\infty \right)=0,\phi \left(0\right)=1.$$

Skin friction coefficients for two models in term of viscosity are

17 $$Cf{Re}^{1/2}=\frac{{\left(1-{\varphi }_{2}\right)}^{-2.5}}{{{\left(1-{\varphi }_{3}\right)}^{2.5}\left(1-{\varphi }_{1}\right)}^{2.5}}\left[\left(1+\alpha \phi (0)\right)F{^{\prime}}{^{\prime}}\left(0\right)+1+\alpha \phi (0)mRe{we}^{2}{\left(F{^{\prime}}{^{\prime}}(0)\right)}^{3}\right],$$

18 $$Cf{Re}^{1/2}=\frac{{\left(1-{\varphi }_{2}\right)}^{-2.5}}{{{\left(1-{\varphi }_{3}\right)}^{2.5}\left(1-{\varphi }_{1}\right)}^{2.5}}\left[{F}^{{^{\prime}}{^{\prime}}}\left(0\right)-\frac{\alpha }{2}{we}^{2}Re{\left(F{^{\prime}}{^{\prime}}(0)\right)}^{3}\right].$$

Concentration rate and heat energy rate are

19 $$-Nu{Re}^{-\frac{1}{2}}=\frac{{K}_{th}}{{K}_{f}}{\theta }^{{^{\prime}}}\left(0\right),$$

20 $$-Sh{Re}^{-\frac{1}{2}}=\frac{{\left(1-{\varphi }_{2}\right)}^{-2.5}}{{{\left(1-{\varphi }_{3}\right)}^{2.5}\left(1-{\varphi }_{1}\right)}^{2.5}}{\phi }^{^{\prime}}\left(0\right).$$

## FEM procedure (Finite element method)

**Figure 2:**
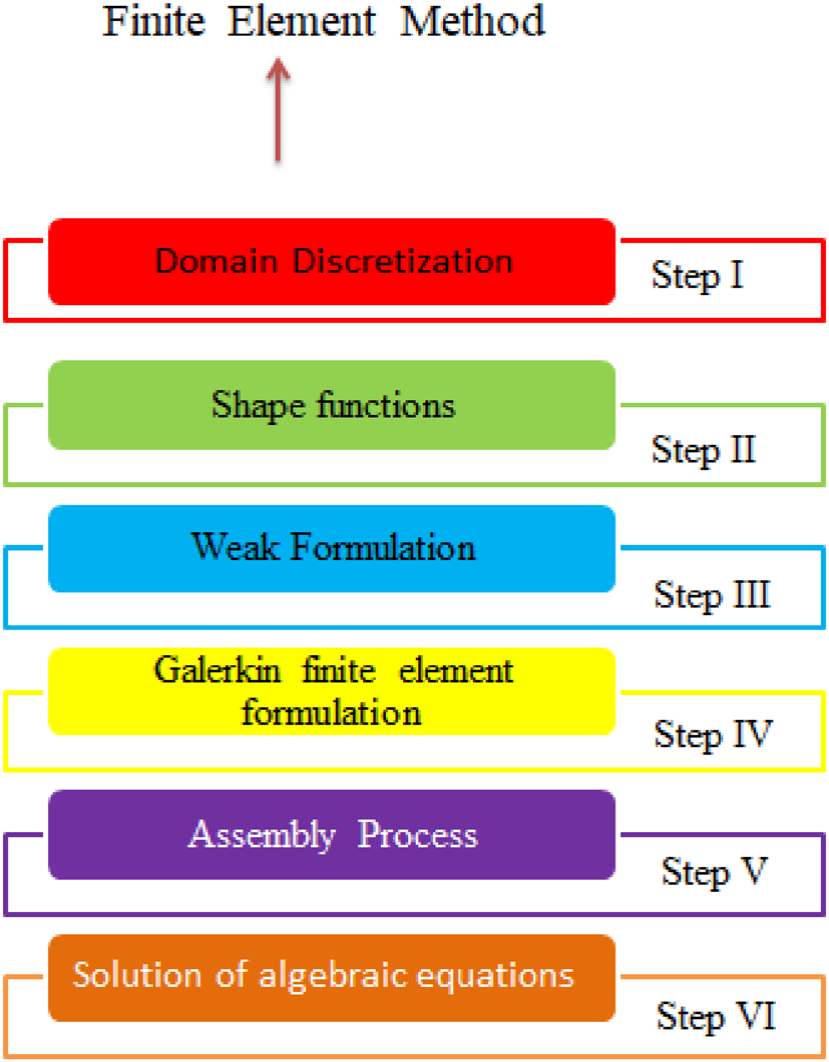
FEM related steps.

Numerical solution of dimensionless ODEs is achieved using finite element method (FEM) which is presented with the help of Fig. 2. Following steps related FEM procedure.

- Desired residuals are known as weighted and residuals are integrated over each elements regarding computational domain;
- The integration process is implemented for development of week form regarding residual equations and mesh free survey is shown through Table 2;
- Stiffness elements are obtained via residual procedure called Galerkin approximations;
- Measure tolerance and error procedure. Table 3 reveals validation of numerical results with published work.
  $$\frac{{\delta }^{i}-{\delta }^{i-1}}{\delta }<{10}^{-5}.$$

**Table 2:** Numerical study of mesh-free for concentration, velocity and heat energy profiles via mid of 300 elements when $\alpha =0.5, m=0.3, Re=4.0, we=3.0, M=3.0, Pr=206, {H}_{t}=-2.0, Sc=0.0, {\delta }_{1}=0.4, ph1=0.003, {\phi }_{1}=0.004, {\phi }_{2}=0.0075, {K}_{c}=-3.0, {\varphi }_{1}=0.003.$

Number of elements	$F{^{\prime}}\left(\frac{{\eta }_{max}}{2}\right)$	$\theta \left(\frac{{\eta }_{max}}{2}\right)$	$\phi \left(\frac{{\eta }_{max}}{2}\right)$
30	0.02294303658	0.5407391166	0.5331705542
60	0.01376586145	0.5245896500	0.5165094221
90	0.01198065198	0.5191518648	0.5109559950
120	0.01124242297	0.5164233899	0.5081778903
150	0.01084212050	0.5147803839	0.5065116330
180	0.01059108809	0.5136848805	0.5054008033
210	0.01041887658	0.5129035893	0.5046079652
240	0.01029345534	0.5123159218	0.5040134244
270	0.01019938656	0.5118566461	0.5035502943
300	0.01012545524	0.5114903521	0.5031791373

**Table 3:** Numerical validation for heat transfer rate when ${\varphi }_{1}=0, {\varphi }_{2}=0, {\varphi }_{3}=0.$

$Pr$	Abel and Mahesha et al.^28^	Present work
1	0.58197	0.5815302043
3	1.16525	1.1655512047
10	2.30800	2.3081502043

## Results and outcomes

Comparative features in term of two viscosity models are studied over a 2D stretchable sheet. A non-Fourier’s approach is considered in energy as well as concentration equation. Heat source and chemical species are addressed. Additionally, suspension of various nanoparticles (silicon dioxide, aluminum oxide and titanium oxide) is inserted in base fluid) termed as ethylene glycol. The productive complex biological model is numerically handled using a finite element approach. Detail reports and discussion are mentioned below.

### Comparison discussion among two viscosity models via fluidic motion

**Figure 3:**
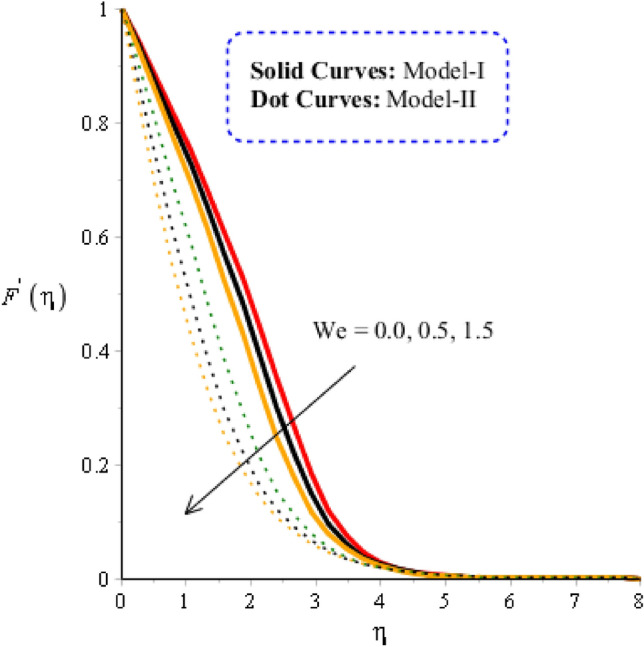
Graphical impact of $We$ on velocity distribution when $\alpha =0.5, m=0.3, Re=4.0, M=3.0, Pr=206,{H}_{t}=-2.0, Sc=0.0, {\delta }_{1}=0.4, {\varphi }_{1}=0.003, {K}_{c}=-2.0, {\varphi }_{2}=0.004, {\varphi }_{3}=0.0075.$

**Figure 4:**
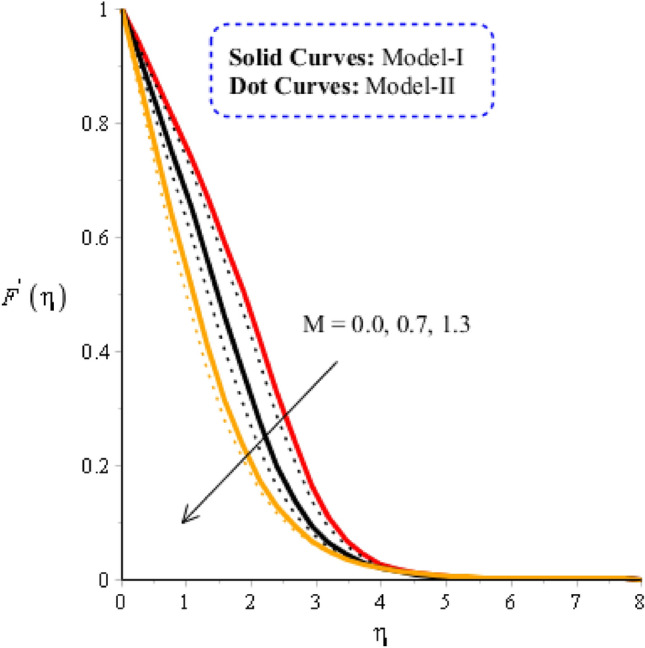
Graphical impact of $M$ on velocity distribution when $\alpha =0.5, m=0.3, Re=4.0, we=3.0, Pr=206,{H}_{t}=-2.0, Sc=0.0, {\delta }_{1}=0.4,{\varphi }_{1}=0.003, {\varphi }_{2}=0.004, {\varphi }_{3}=0.0075, {K}_{c}=-3.0.$

**Figure 5:**
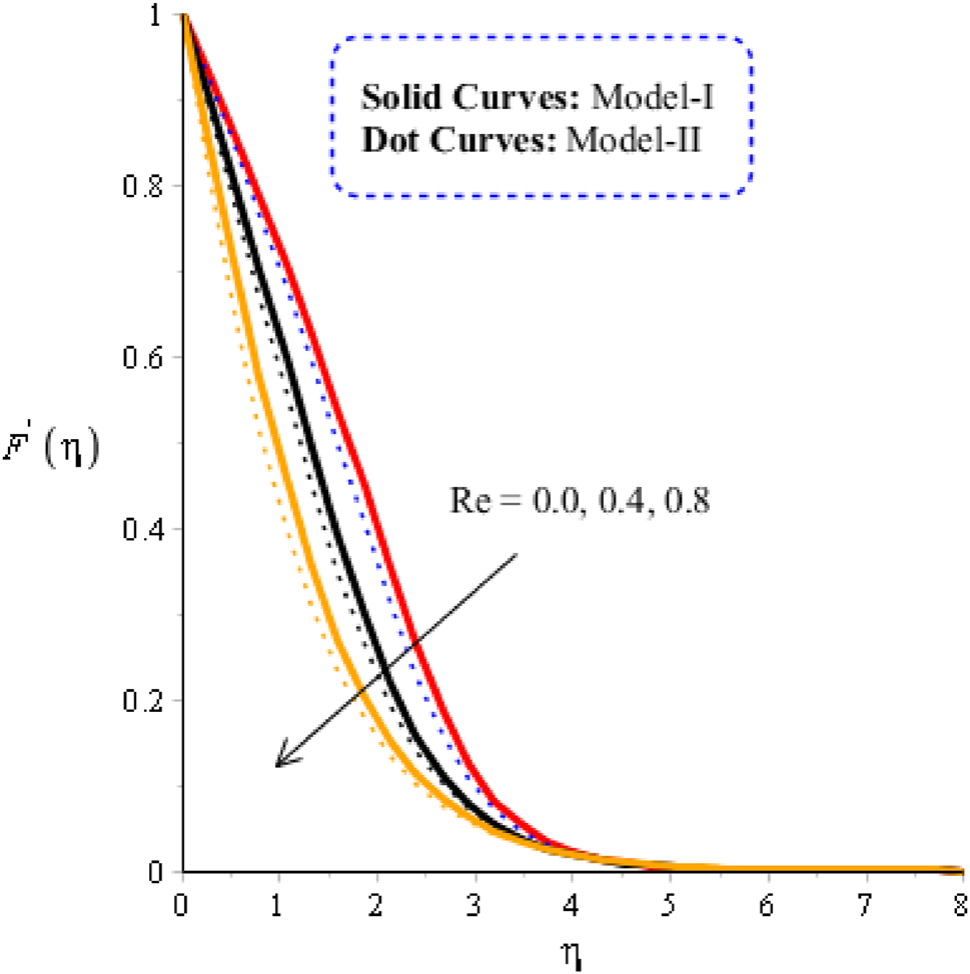
Graphical impact of $Re$ on velocity distribution when $\alpha =0.5, m=0.3, we=3.0, M=3.0, Pr=206,{H}_{t}=-2.0, Sc=0.0, {\delta }_{1}=0.4, ph1=0.003, {\varphi }_{1}=0.003, {\varphi }_{2}=0.004, {\varphi }_{3}=0.0075, {K}_{c}=2.0.$

**Figure 6:**
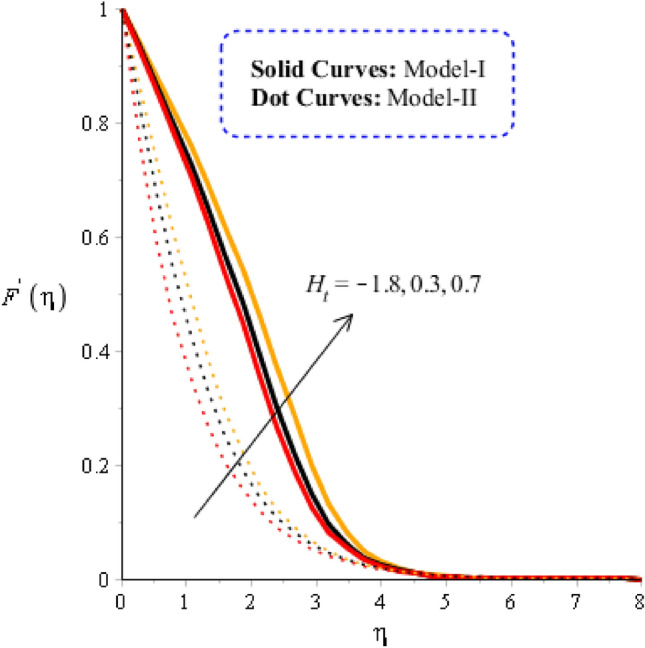
Graphical impact of ${H}_{t}$ on velocity distribution when $\alpha =0.5, m=0.3, Re=4.0, we=3.0, M=3.0, Pr=206, {K}_{c}=3.0, Sc=0.0, {\delta }_{1}=0.4, {\varphi }_{1}=0.003, {\varphi }_{2}=0.004, {\varphi }_{3}=0.0075.$

In the current subsection, acceleration into fluidic particles is measured in Figs. 3, 4, 5 and 6, including two viscosity models and tri-hybrid nanomaterial. It is mentioned that solid curves are captured to determine the impact of acceleration and fluidic motion for model-I while model-II is represented by dot curves. Figure 3 is plotted to visualize the role of Weissenberg number on fluidic motion, including two viscosity models. The acceleration into fluidic particles is produced less when Weissenberg number is enhanced. This is because Weissenberg number is ratio among elastic force and viscous force. From physically, Weissenberg number has inversely proportional relation against viscous force. Therefore, viscosity of fluid is increased when Weissenberg number is increased. Further, the concept of ${\varvec{w}}{\varvec{e}}$ is utilized into motion due to rheology of synovial fluid. Physically, ${\varvec{w}}{\varvec{e}}$ has inverse relation versus viscous force. Therefore, the thicknesses associated momentum layers are declined when ${\varvec{w}}{\varvec{e}}$ is enhanced. Thickness for momentum layers for ${\varvec{w}}{\varvec{e}}=0$ is higher than thicknesses for ${\varvec{w}}{\varvec{e}}\ne 0$. Figure 4 is plotted to observe visualization of flow analysis versus magnetic parameter. Flow becomes decrease when Lorentz force is implemented. It is mentioned that appearance of magnetic parameter is produced using concept of Lorentz force. Moreover, flow experiences less Lorentz force for model-II rather than flow for the case of model-II. Thickness linked with momentum boundary layer for model-II is less than Thickness linked with momentum boundary layer for model-I. Physically, the flow is reduced because of negative Lorentz force. The strength of magnetic field is applied against flow behavior. Therefore, resistance during flow is generated when Lorentz force is utilized. Hence, Lorentz force is utilized as reducing role into flow and momentum boundary layers thickness. The role of Reynolds number along with two models (viscosity models) is noticed on velocity distribution in Fig. 5. It is estimated that flow develops slow down against higher values of Reynolds number. It is occurred because Reynolds parameter is ratio among viscous force and inertial force. Hence, viscous force is increased versus increment in Reynolds number. Additionally, the flow is generated significantly for mode-I rather than flow is generated for model-II. Figure 6 reveals the behavior of heat source (${{\varvec{H}}}_{{\varvec{t}}}$) on velocity profile. Flow becomes maximize using concept of higher values of ${{\varvec{H}}}_{{\varvec{t}}}$. The concept of ${{\varvec{H}}}_{{\varvec{t}}}$ on flow behavior is produced when external thermal energy source is utilized at wall. Two types phenomena are experienced on flow based on heat generation and heat absorption. Heat generation is happened for ${{\varvec{H}}}_{{\varvec{t}}}>0$ while heat absorption for ${{\varvec{H}}}_{{\varvec{t}}}<0.$

### Comparison discussion among two viscosity models via fluidic thermal energy

**Figure 7:**
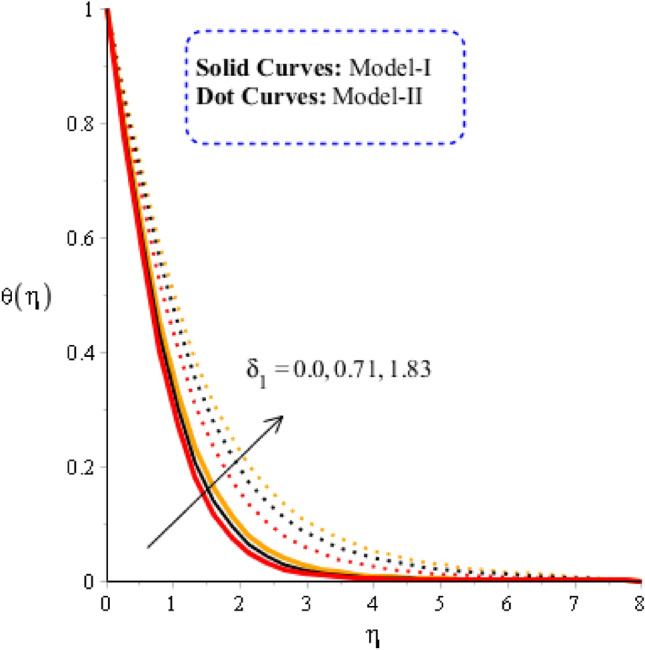
Graphical impact of ${\delta }_{1}$ on thermal distribution when $\alpha =0.5, m=0.3, Re=4.0, we=3.0, M=3.0, Pr=206,{H}_{t}=-2.0, Sc=0.0, {\varphi }_{1}=0.003, {\varphi }_{2}=0.004, {\varphi }_{3}=0.0075, {K}_{c}=-2.0.$

**Figure 8:**
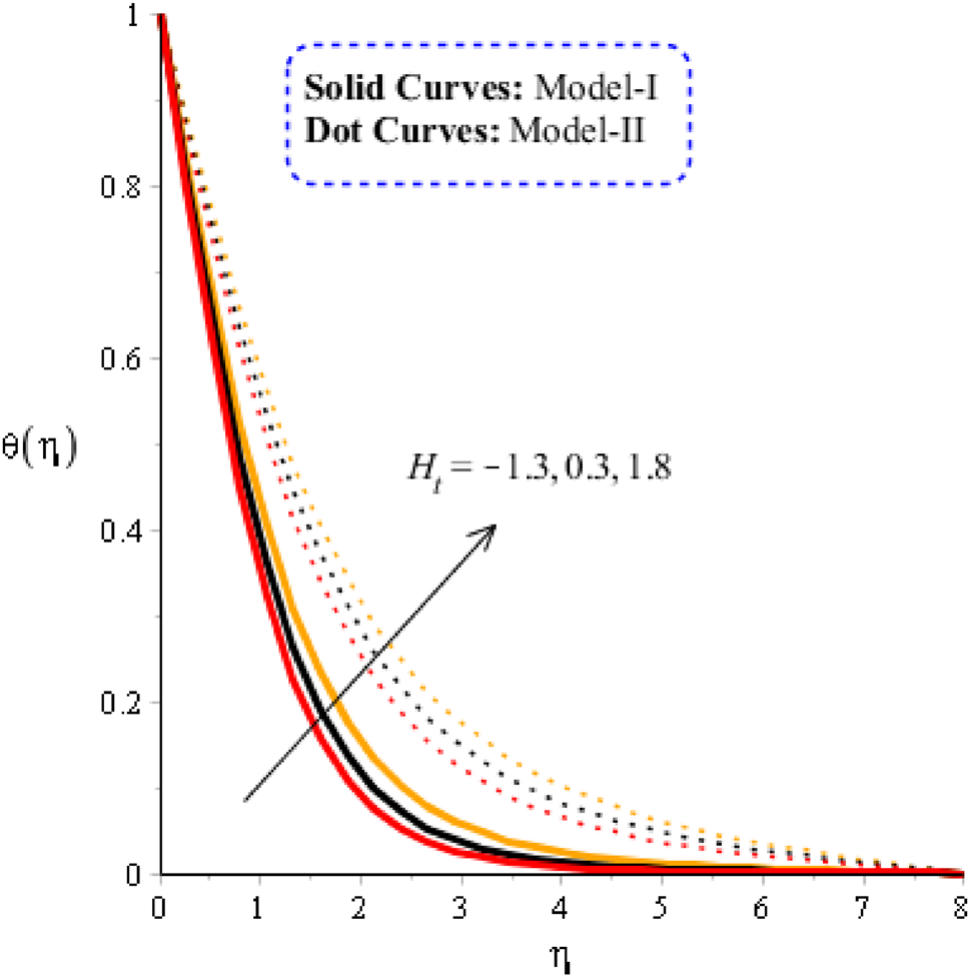
Graphical impact of ${H}_{t}$ on thermal distribution when $\alpha =0.5, m=0.3, Re=4.0, we=3.0, M=3.0, Pr=206, {K}_{c}=-3.0, Sc=0.0, {\delta }_{1}=0.4, {\varphi }_{1}=0.003, {\varphi }_{2}=0.004, {\varphi }_{3}=0.0075.$

**Figure 9:**
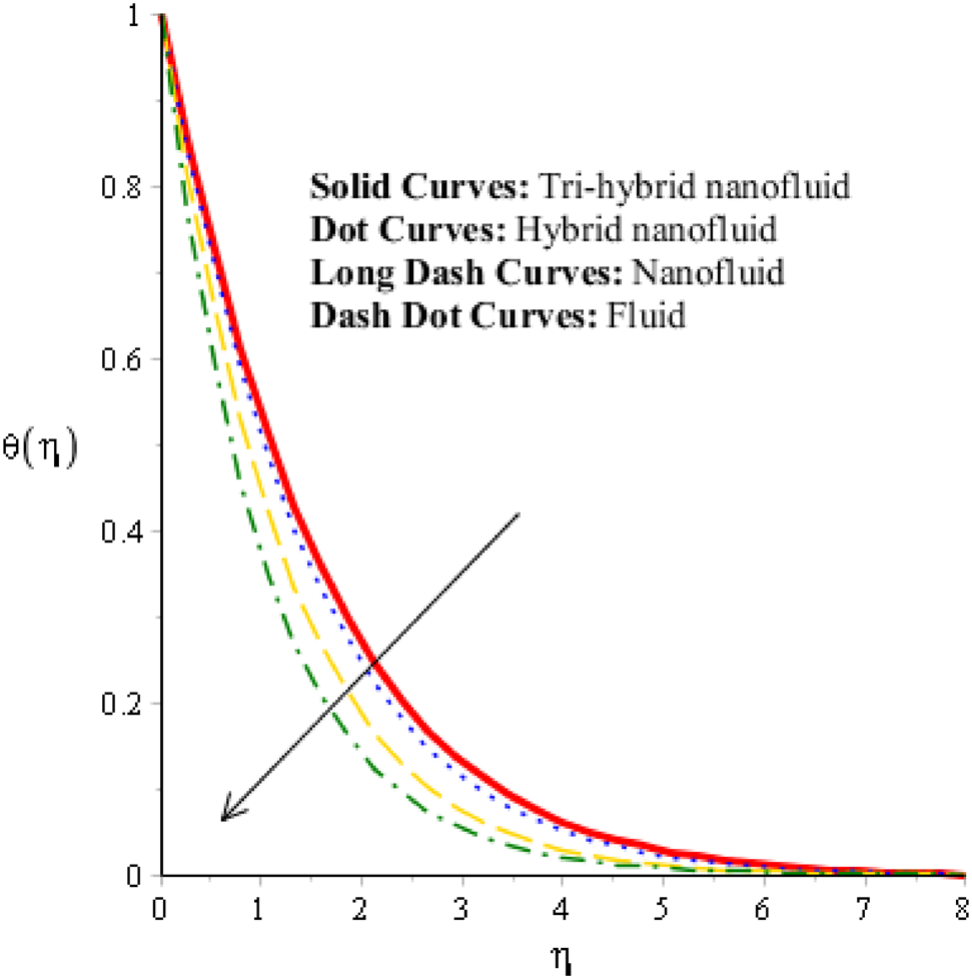
Comparative achievement among fluid, hybrid nanofluid, nanofluid and tri-hybrid nanofluid when $\alpha =0.5, m=0.3, Re=4.0, we=3.0, M=3.0, {K}_{c}=-3.0, Pr=206,{H}_{t}=-2.0, Sc=0.0, {\varphi }_{1}=0.003, {\varphi }_{2}=0.004, {\varphi }_{3}=0.0075.$

The comparative thermal features are estimated versus time relaxation, heat source parameters for model-I and model-II considering in Figs. 7 and 8. Figure 7 is captured to estimate the behavior of time relaxation number on temperature profile. It is determined that maximum heat energy is achieved for the case of ${\gamma }_{1}$. The maximum heat energy is experienced when time relaxation number is enhanced. This is occurred maximum restoration regarding heat energy is produced. Representation of ${\delta }_{1}$ is utilized in energy equation due to non-Fourier’s approach. It is dimensionless parameter based on non-Fourier’s law and ${\delta }_{1}$ makes ability to restore heat energy into particles. Further, maximum manufacture of heat energy for model-II as compared for the case of model-I. Hence, thickness linked with thermal layers is higher for model-II rather than for model-I. Figure 8 captures the observation of heat source number on thermal profile. Thermal energy is gained maximum when heat source parameter is implemented. Two type’s phenomena are experienced on flow based on heat generation and heat absorption. Heat generation is happened for ${\mathrm{H}}_{\mathrm{t}}>0$ while heat absorption for ${\mathrm{H}}_{\mathrm{t}}<0.$ It can be noticed that heat generation process is higher than heat absorption process. Thermal layers can be managed trough variation in ${\mathrm{H}}_{\mathrm{t}}.$ Physically, it can be noticed that thermal layers in term of thickness are increased versus increment values of heat source number. Figure 9 is observed as significant in term of comparison among tri and hybrid nanomaterial. Solid lines are captured to comparative visualization among fluid, nanofluid, hybrid nanomaterial and tri-hybrid nanomaterial in base fluid. It is estimated that heat energy in view of tri-hybrid nanomaterial is higher than thermal energy for case hybrid nanomaterial, fluid and nanofluid. Hence, maximum enhancement and cooling process are achieved for case of ternary hybrid nanofluid.

### Comparison discussion among two viscosity models via fluidic mass species

**Figure 10:**
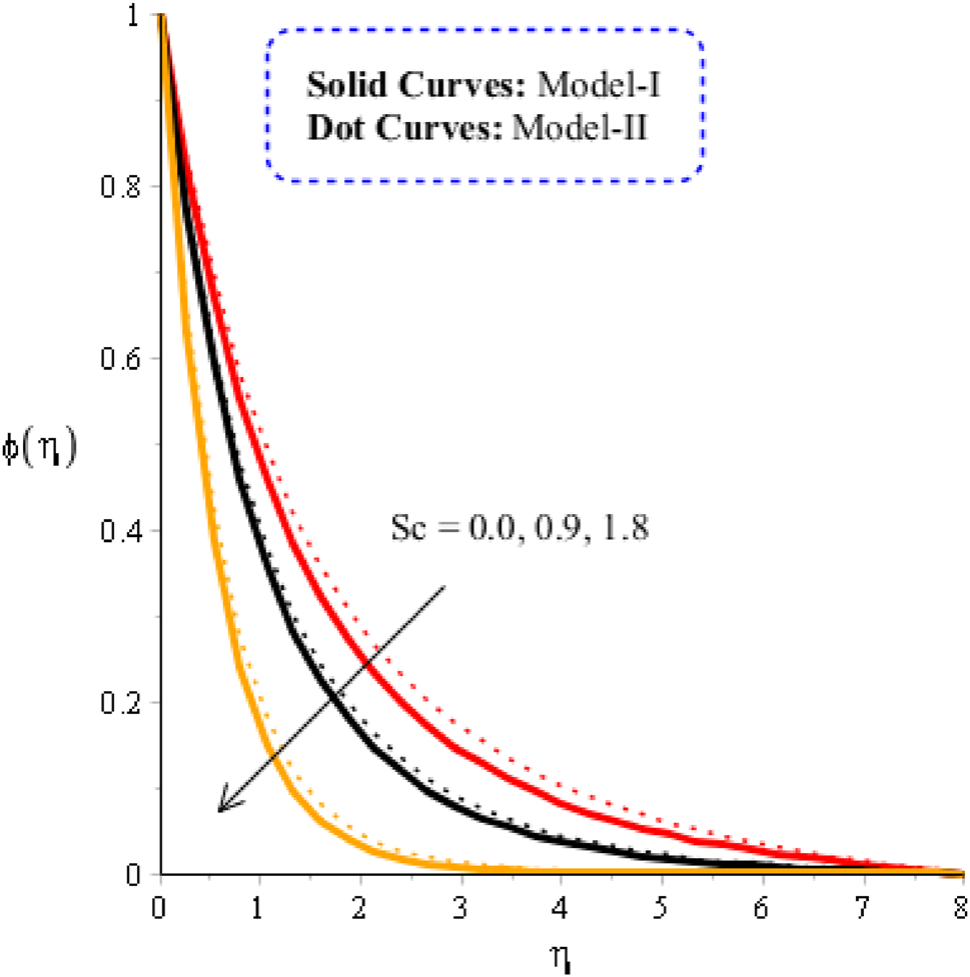
Graphical impact of $Sc$ on concentration distribution when $\alpha =0.5, m=0.3, Re=4.0, we=3.0, M=3.0, Pr=206,{K}_{c}=-3.0, {H}_{t}=-2.0, Sc=0.0, {\varphi }_{1}=0.003, {\varphi }_{2}=0.004, {\varphi }_{3}=0.0075.$

**Figure 11:**
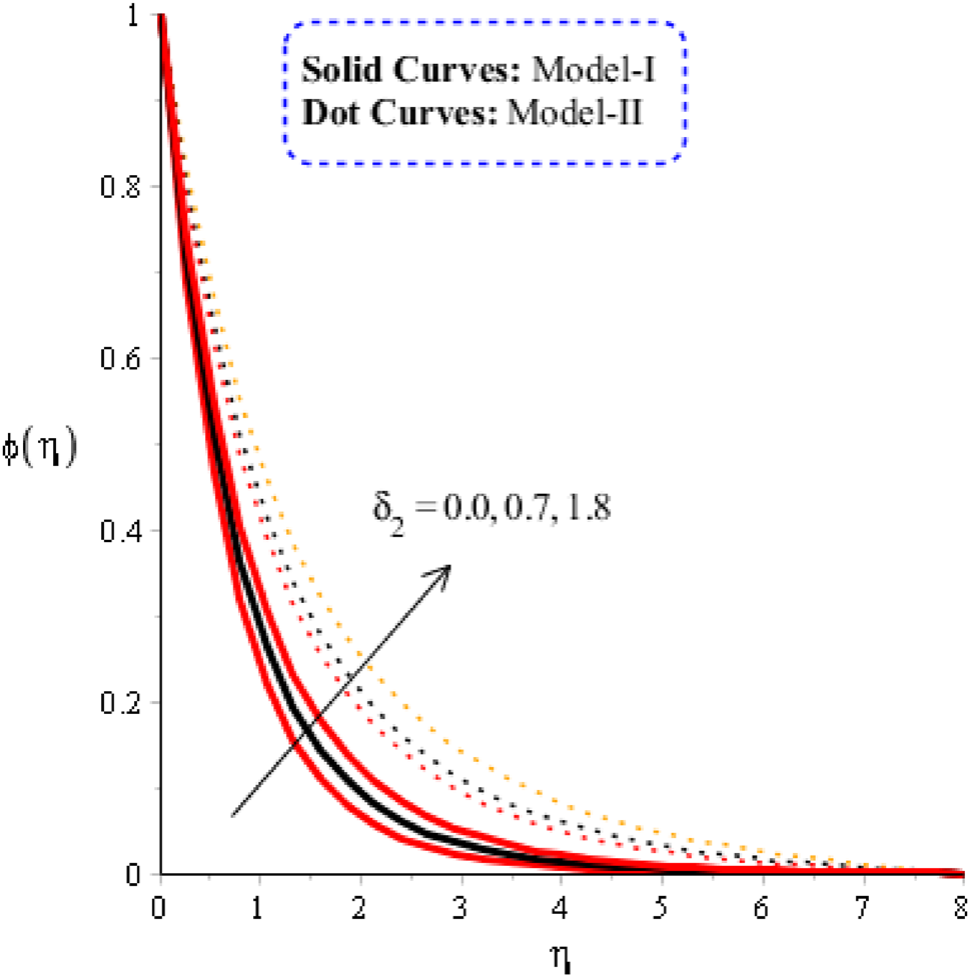
Graphical impact of ${\delta }_{2}$ on concentration distribution when $\alpha =0.5, m=0.3, Re=4.0, we=3.0, M=3.0, Pr=206,{{K}_{c}=-3.0, H}_{t}=-2.0, Sc=0.0, {\delta }_{1}=0.4, {\varphi }_{1}=0.003, {\varphi }_{2}=0.004, {\varphi }_{3}=0.0075.$

**Figure 12:**
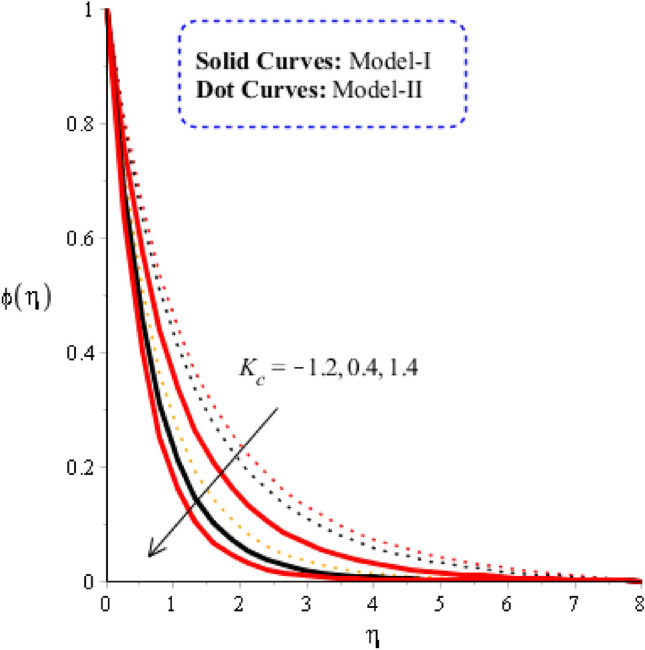
Graphical impact of ${K}_{c}$ on concentration distribution when $\alpha =0.5, m=0.3, Re=4.0, we=3.0, M=3.0, Pr=206,{H}_{t}=-2.0, Sc=0.0, {\delta }_{1}=0.4, {\varphi }_{1}=0.003, {\varphi }_{2}=0.004, {\varphi }_{3}=0.0075.$

Figures 10, 11 and 12 are prepared to determine the observations of mass diffusion against distribution in Schmidt number, chemical reaction number and time relaxation number. Figure 10 is prepared to determine estimation of Schmidt number including model-I and model-II. Diffusion into solute particles becomes slow down when $Sc$ is enhanced. Schmidt parameter is defined as ratio among momentum and mass species into fluid particles. So, an inversely proportional trend is noticed against change in mass species. Consequently, the diffusion of species becomes slowdown when $Sc$ is increased. Production of mass diffusion for $Sc=0$ is greater than production of mass diffusion for $Sc\ne 0.$ Thickness related thermal layers are declined versus variation in Schmidt number. The role of time relaxation parameter on mass diffusion for model-I and model-II is considered in Fig. 11. The diffusion into mass species is declined when time relaxation parameter is decreased. Moreover, the mass diffusion for model-II is greater than for the case model-I. It is observed that occurrence of ${\delta }_{2}$ is noticed using theory of non-Fourier’s in concentration equation. Diffusion of mass species is declined when theory of non-Fourier is utilized. Figure 12 is prepared to estimate the variation in mass diffusion when chemical reaction number in the presence of model-II and model-I. Figure 12 reveals the process of generative chemical reaction (${K}_{c}<0$) is less than process of destructive chemical reaction (${K}_{c}>0$). Appearance of ${K}_{c}$ is modeled when chemical reaction is occurred into mass species. Transport of mass species is reduced for model-II and model-I when chemical reaction number is enhanced.

### Impacts of Sherwood number, skin friction coefficient and heat transfer rate

Impacts of Sherwood number, heat transfer rate and skin friction coefficient are predicted versus variation in Weissenberg, heat source, magnetic and Schmidt numbers. These visualizations are recorded in Table 4. It is noticed that divergent velocity (skin friction coefficient) and heat transport rate are enhanced versus the variation in heat source number. But constant behavior for mass diffusion rate is observed for variation in heat source number. Magnetic parameter brings declination into heat transfer and mass diffusion rated while divergent velocity is decreased. Additionally, mass diffusion rate is enhanced when Schmidt number is increased. In case of Weissenberg number, flow rate is decreased and mass diffusion rate and heat energy rate are increased.

**Table 4:** Numerical consequences of mass diffusion rate, divergent velocity and heat energy rate versus impacts of heat source, Weissenberg, Schmidt and magnetic parameters when $\alpha =0.5, m=0.3, Re=4.0, Pr=206, {\delta }_{1}=0.4, ph1=0.003, {\varphi }_{1}=0.003, {\varphi }_{2}=0.004, {\varphi }_{3}=0.0075.$

Change in parameters		$-Cf{Re}^\frac{1}{2}$	$-Nu{Re}^{-\frac{1}{2}}$	$-Sh{Re}^{-\frac{1}{2}}$
${H}_{t}$	− 1.8	0.5819082531	1.704545484	0.1251079132
	0.7	0.5509136062	1.696530334	0.1251079132
	1.4	0.5240101703	1.685344690	0.1251079132
$we$	0.0	0.5949186967	1.631731458	0.1251079132
	0.7	0.5819212062	1.641203201	0.1281250122
	1.3	0.5631126602	1.670330253	0.1290020237
$Sc$	0.0	0.5949186967	1.691723859	0.1249999995
	0.3	0.5949186967	1.722068872	0.5886630903
	0.7	0.5949186967	1.740594028	0.9412612184
$M$	0.0	0.7621803004	1.693933394	0.1253751105
	0.01	0.7852799081	1.684292106	0.1233774407
	0.08	0.8216289994	1.664644057	0.1213796206

## Conclusions

Comparison thermal aspects and solute aspects among model-I and model-II including tri-hybrid nanomaterial are observed past stretching frame. Heat energy and diffusion into mass species are carried out by non-Fourier’s approach. Additionally, heat source and chemical species are implemented. Such complex problem is developed and numerical tackled by finite element approach. Main finding of such complex problem are mentioned below.

- Velocity profile is declined using higher numerical values of magnetic field intensity based on Lorentz force.
- Velocity profiles for model-I have less magnitudes rather than magnitudes for velocity profiles for model-II.
- External heat source brings enhancement into thermal energy and flow behavior.
- Cooling process thermal process can be achieved significantly by using mechanism of ternary hybrid nanofluid rather than mechanism of hybrid nanofluid.
- Production of mass species and thermal energy for case of model-II as compared for model-I.
- Mass diffusion decreases versus impacts of chemical reaction number and Schmidt number.
- Current development is applicable in biological field which is used in condyloid joints, hinge joints, pivot joints.
- Such complex analysis is applicable in biological field which is used in condyloid joints, hinge joints, pivot joints and shoulder joints.

## Data Availability

The datasets used and/or analysed during the current study available from the corresponding author on reasonable request.

## List of symbols

$$v,u$$: Velocity components
$\rho$: Density of fluid
$m$: Power law index number
$$\gamma $$: Material number
$$\sigma $$: Electrical conductivity
$${\gamma }_{1}, {\gamma }_{2}$$: Relaxation parameters
$T, {T}_{\infty }$: Temperature and ambient temperature
$$K$$: Thermal conductivity
$D, {K}_{c}$: Mass diffusion and chemical reaction number
$th$: Tri-hybrid nanofluid
$${T}_{w}$$: Wall temperature
$$\psi $$: Stream function
$$\nu $$: Kinematic viscosity
$$hnf$$: Hybrid nanofluid
$$we$$: Weissenberg number
$$Pr$$: Prandtl number
${H}_{t}$: Heat source number
EG: Ethylene glycol
$$Ti{O}_{2}$$: Titanium oxide
$Cf$: Skin friction coefficient
$$Nu$$: Nusselt number
$$Y,X$$: Space coordinates
$\mu$: Viscosity of fluid
$\alpha$: Viscosity parameter (concentration dependent)
$$nf$$: Nanofluid
${B}_{0}$: Magnitude of magnetic field
${Q}_{0}$: Heat source/absorption
${C}_{p}$: Specific heat capacitance
${k}_{1}$: Chemical reaction number
${C}_{\infty }, C$: Ambient concentration and concentration
$$\infty $$: Infinity number
$${C}_{w}$$: Wall concentration
$$\eta $$: Independent variable
$${\varphi }_{2}, {\varphi }_{1}, {\varphi }_{3}$$: Volume fractions
ODEs: Ordinary differential equations
$$Re$$: Reynolds number
$$M$$: Magnetic number
$${\delta }_{1}, {\delta }_{2}$$: Relaxation numbers
$$Sc$$: Schmidt number
$$Si{O}_{2}$$: Silicon dioxide
$${Al}_{2}{O}_{3}$$: Aluminum oxide
$$Re$$: Reynolds number
$$Sh$$: Sherwood number
